# Weight Trajectories During Inpatient Treatment for Anorexia Nervosa: A Dynamic Time Warp Analysis

**DOI:** 10.1002/eat.24573

**Published:** 2025-10-15

**Authors:** Marianne Tokic, Georg Halbeisen, Karsten Braks, Thomas J. Huber, Nina Timmesfeld, Georgios Paslakis

**Affiliations:** ^1^ Department of Medical Informatics, Biometrics and Epidemiology Ruhr‐University Bochum Bochum Germany; ^2^ University Clinic for Psychosomatic Medicine and Psychotherapy, Medical Faculty, Campus East‐Westphalia Ruhr‐University Bochum Luebbecke Germany; ^3^ Centre for Eating Disorders Klinik Am Korso Bad Oeynhausen Germany

**Keywords:** anorexia nervosa, dynamic time warping, inpatient treatment, psychotherapy, weight change

## Abstract

**Background:**

Restoring weight is a primary goal during anorexia nervosa (AN) treatment. Previous studies linked different weight gain profiles to treatment outcomes, but there is currently no consensus on profile shapes and numbers. We argue that heterogeneity stems from temporal distortions (“warping”) in weight gain, and that similar weight improvements can stretch over different time periods. We thus favor a novel non‐parametric solution that accounts for warping to identify weight trajectories.

**Method:**

Time series clustering with dynamic time warping (DTW) was used to identify weight change trajectories among *N* = 518 patients with AN during inpatient treatment. Within‐person body‐mass‐index gain (∆ BMI) served as our primary dependent variable to identify clusters. We characterized clusters based on admission psychopathology scores, and analyzed associations of cluster affiliation with changes in clinical outcomes between admission and discharge using linear and logistic models.

**Results:**

We identified four distinct clusters, with *n* = 76 patients showing initial weight gain (Cluster 1), *n* = 329 showing continuous weight gain (Cluster 2), *n* = 70 showing initial weight loss and recovery (Cluster 3), and *n* = 43 showing weight loss (Cluster 4). The four clusters differed in terms of admission BMI, psychopathology scores, and days spent in treatment, and cluster assignment predicted treatment outcomes.

**Conclusion:**

Using one of the largest hitherto examined samples for weight gain profile analysis, the novel DTW‐based approach provided an overall more elaborated set of outcome‐predictive profiles compared to previous studies, which could help inform individualized treatment strategies and allocate therapeutic resources efficiently.

**Summary**

- Underweight in patients with anorexia nervosa is a major health concern and is associated with high mortality rates. This study used a novel statistical approach to identify similarities in weight gain patterns among a large group of these patients.
- The results may ultimately help to improve treatment for anorexia nervosa.

**Abbreviations**
AN: anorexia nervosa
BDI: Beck depression inventory
BMI: body‐mass‐index
COP: context‐independent optimality and partiality
DB: Davies–Bouldin index
DTW: dynamic time warping
ED: eating disorders
EDE‐Q: eating disorder examination‐questionnaire
ICD: international statistical classification of diseases and related health problems

## 1 Background

Anorexia nervosa (AN) is a severe mental disorder characterized by significant weight loss or failure to gain weight appropriately for age due to restrictions in energy intake, an intense fear of weight gain, and a disturbed body image (American Psychiatric Association 2013). Its variants include a restricting subtype and a binge‐eating/purging subtype that presents with binge‐eating or purging episodes (e.g., self‐induced vomiting). Although AN is comparatively rare with a lifetime prevalence of 1.4% in women and 0.2% in men (Galmiche et al. 2019), it ranks among the most lethal mental disorders (Cuntz et al. 2023). Partly due to severe underweight, patients with AN have a 5.9 times higher all‐cause mortality risk compared to the general population (Chesney et al. 2014). Restoring weight is thus a primary goal in AN treatment (Herpertz‐Dahlmann et al. 2015).

Identifying and understanding heterogeneity in weight restoration is of vital importance (Garber et al. 2016; Halbeisen, Amin, et al. 2024). Indeed, the amounts and trajectories of weight gain during AN treatment can vary considerably between patients. Evidence‐based guidelines recommend between 500 and 1500 g weight gain per week during inpatient treatment (Hilbert et al. 2017), but significantly lower weekly weight gains are observed even in tightly controlled settings (Zeeck et al. 2018). Moreover, while some patients may gain weight at a constant rate, that is, linearly during treatment, others may gain weight only initially, at later stages, stagnate at some point, or even lose weight to the extent that it offsets previous gains (Di Lodovico et al. 2024; Lebow et al. 2019). Since weight gain features such as faster weight gain during early treatment have been linked to improved weight and reduced psychopathology at discharge and follow‐up (Austin et al. 2021; Halbeisen, Amin, et al. 2024; Kolar et al. 2022; Le Grange et al. 2014; Wales et al. 2016), correctly identifying and characterizing weight gain trajectories has the potential to predict treatment outcomes and possibly improve and personalize AN treatment (Makhzoumi et al. 2017).

### 1.1 Current Approaches for Characterizing Weight Trajectories

Previous studies thus far used (growth) mixture modeling to identify weight gain profiles in patients with AN. Mixture modeling describes a broad class of statistical approaches that assume data are composed (i.e., a mixture) of several subpopulations with different response patterns (called “classes”), for example, with similar progressions of weight over time (“growth curves”), and attempt to discern these patterns retrospectively in a way that maximizes within‐class homogeneity and between‐class heterogeneity (Prince and Fidler 2021).

However, contrasting promises of reducing the complexity of individual‐level weight gain, mixture modeling has led to heterogeneous results regarding the shapes and number of weight gain profiles. For example, both Makhzoumi et al. (2017) and Berona et al. (2018) distinguished between three classes of fast, moderate, and slow weight gain among 211 adults with AN over 60 days inpatient treatment and 102 adults over 30 days of treatment, respectively. Jennings et al. (2018), instead, characterized weight trajectories among 500 inpatients with AN into weight gain, weight plateau (i.e., only initial weight gain), weight fluctuation, and treatment‐resistant (i.e., weight loss) groups. Four‐class solutions were also found in Wade et al. (2021) and Di Lodovico et al. (2024); however, the former distinguished between fast weight gain, moderate weight gain, and two groups of no improvement among 120 patients over 13 outpatient sessions, whereas the latter identified four different improvement‐only profiles among 181 patients over 5 months of treatment (i.e., late‐rising‐flattening, late‐rising‐steady, early‐rising‐flattening, and early‐rising‐steady groups). Finally, Lebow et al. (2019) distinguished five distinct profiles in age‐standardized BMI trajectories among 153 adolescents over 6 months of inpatient treatment, i.e., slow‐but‐steady improvements, moderate gains with mid‐treatment maintenance, “dramatically” rapid gains with late stabilization, rapid gains with early stabilization, and weight maintenance. Thus, there is currently no clear consensus on how to characterize weight gain profiles in patients with AN.

### 1.2 “Warped” Timing Dimensions May Obscure Similarity Detection

There are several potential reasons for the differences in estimated weight gain profiles in previous investigations. Weight gain profiles and their total number might differ based on features of the intervention protocol and setting (e.g., inpatient vs. outpatient; Makhzoumi et al. 2017; Wade et al. 2021), features of the population (e.g., age, or whether patients with atypical AN symptoms were included; Lebow et al. 2019), the overall length of the study period (e.g., 30 days vs. 5 months; Berona et al. 2018; Di Lodovico et al. 2024), and whether or not absolute weight gains or weight gains relative to admission were considered. Except for Jennings et al. (2018), most studies also included samples close to or less than 200 patients, which may lead to instability in trajectory estimation (Jaki et al. 2019). However, it is important to note that previous studies, inherent to their estimation methods, also presumed that weight changes within subpopulations progress similarly within fixed units of time (be it in a linear or non‐linear fashion), which could interfere with the detection of subpopulation trajectories if similar patterns unfold over varying (warped) timing dimensions (Franses and Wiemann 2020).

**FIGURE 1:**
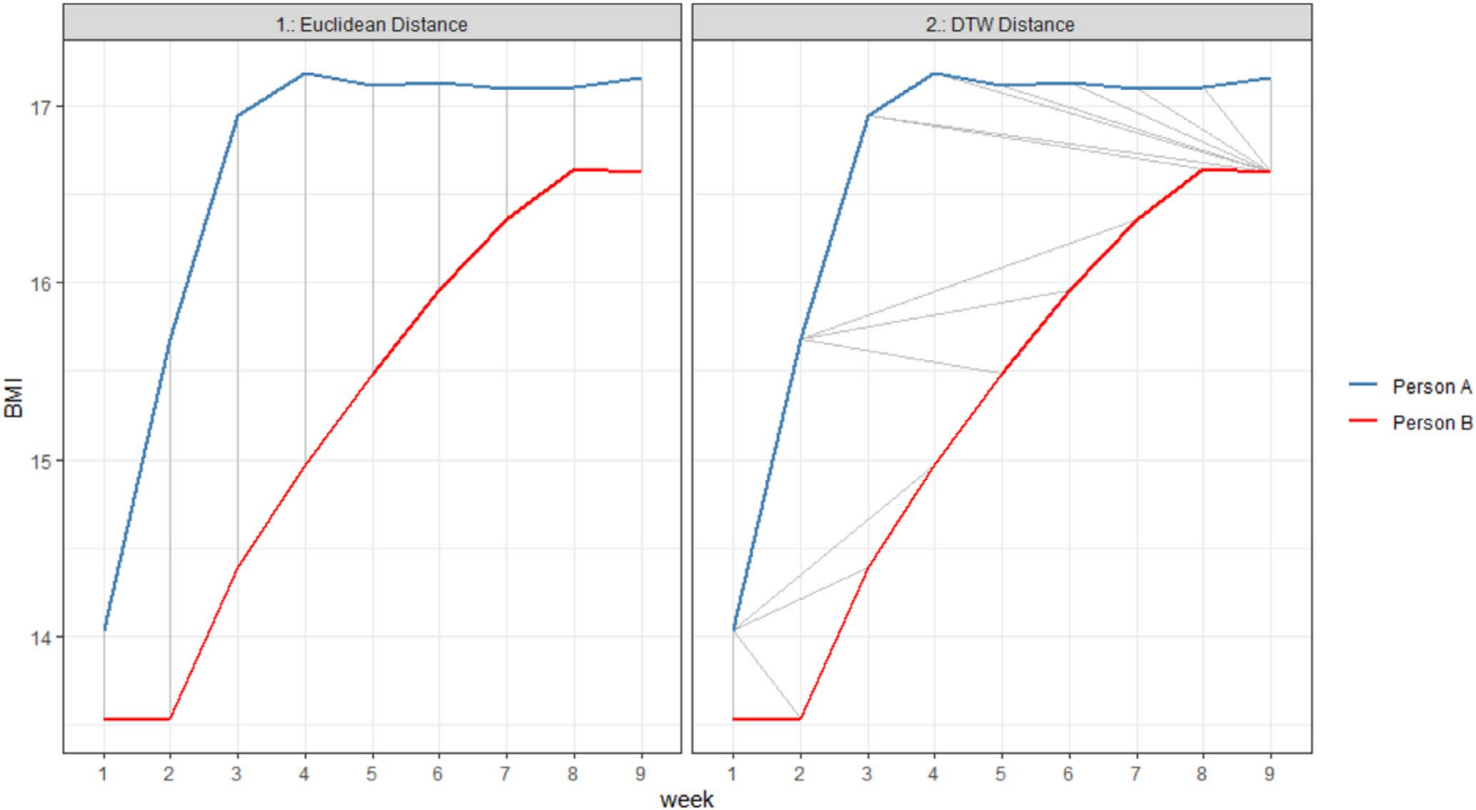
Visual comparison of matched weights of two hypothetical patients based on DTW‐based (right side) vs. time‐based (left side) distances. DTW allows us to compare the shape of trajectories even if they start at different time points and/or move at different speeds. Visualization code adapted from Sobolewska (2018).

Consider, for example, two hypothetical patients with AN who show rapid weight gains at the start of their treatment, which has been associated with improved clinical outcomes (Halbeisen, Amin, et al. 2024; Kolar et al. 2022). One patient might show pronounced weight gains during weeks one to three, whereas the other patient might show a similar pattern of improvement starting in week two and continuing until week seven (see Figure 1). However, because there is a delay in the onset of the weight change pattern, and because one is stretched over three more weeks than the other, there is a chance these patients might be assigned to different latent subpopulations when comparing weight gains strictly by the time of measurement despite their (clinically relevant) similarity. Thus, we believe it is important to account for temporal distortions when identifying and characterizing weight gain trajectories during AN treatment.

A novel non‐parametric solution for examining similarities in temporally distorted time series is time series clustering with Dynamic Time Warping (DTW; Aghabozorgi et al. 2015; Sardá‐Espinosa 2019). DTW refers to an algorithm that calculates the distances between data points of shifted time series (i.e., time series that start at different points or stretch over different periods) more accurately than traditional (i.e., Euclidean) distance measures (Cuturi and Blondel 2017). DTW allows us to compare the shape of trajectories that start at different moments in time or move at different speeds (as illustrated in Figure 1). DTW was initially developed for speech recognition (Sakoe and Chiba 1978) but has recently been applied to study symptom trajectories in health domains (Booij et al. 2021; Doornenbal and Bakx 2021; Mesbah et al. 2024). However, to the best of our knowledge, only two studies employed a DTW algorithm in the context of eating disorders (Kopland et al. 2024; Slof‐Op't Landt et al. 2022), and none thus far used DTW‐based clustering to analyze weight trajectories in patients with AN. Given its unique ability to detect similar shapes of trajectories in time series with “warped” timing dimensions, DTW‐based time series clustering is an innovative approach that could help to identify weight gain trajectories more reliably and potentially explain some of the heterogeneity across previous findings.

## 2 Method

### 2.1 Aim, Design and Setting of the Study

In the present study, we used DTW‐based time series clustering to identify and characterize individual‐level weight change trajectories among a large sample of patients with AN during inpatient treatment. Specifically, we used routine admission data from an eating disorder specialty clinic to examine the weight gain trajectories of patients with AN who completed a significant amount of the treatment program (i.e., the clinic's average length of inpatient stay is 42 days). Based on previous literature, we expected to find between three and five weight gain trajectories, including those of early weight gain (Halbeisen, Amin, et al. 2024); identifying their specific number and shape remained our primary target. Because differences in weight gain profiles have been linked to patients' clinical outcomes and the potential to individualize treatment regimens, we further explored whether specific patients' admission characteristics would be linked to different DTW‐based clusters and whether clustering, in turn, would predict differences in weight gain and psychopathology outcomes at discharge.

### 2.2 Sample

We included the clinical records of *N* = 518 patients (504 women, 14 men; *M*_age_ = 21.8, age range: 13–66 years; *M*_BMI_ = 15.9 kg/m^2^) with binge‐eating/purging AN (*n* = 210) or restricting AN (*n* = 308), diagnosed by experienced clinicians according to the International Statistical Classification of Diseases and Related Health Problems, 10th Revision (ICD‐10) criteria. These patients were part of a large cohort of more than 2000 patients with eating disorders originally described elsewhere (Halbeisen, Braks, et al. 2022). We only included patients with AN treated for at least 42 days (i.e., the clinic's average length of inpatient stay), with weighings for at least 4 weeks, and complete psychopathology admission and discharge assessments. The retrospective analyses were approved by the Ethics Committee of the Ruhr‐University Bochum's Medical Faculty at Campus East‐Westphalia (AZ 2021‐849). Datasets are available from the corresponding author upon reasonable request.

### 2.3 Measures and Procedure

All patients were admitted to a specialty eating disorder facility in Germany between January 2018 and December 2021. Inpatient treatment followed a multimodal rehabilitation concept based on psychodynamic and cognitive‐behavioral approaches and included individual and group psychotherapy, psychoeducation, nutritional rehabilitation, and complementary therapies. Therapists were physicians and psychologists participating in regular supervision.

Patients completed a sociodemographic questionnaire at admission (e.g., age, sex) (Traut et al. 2023) and measures of ED‐specific and general psychopathology at admission and discharge. Specifically, they completed the eating disorder examination‐questionnaire (EDE‐Q; Hilbert et al. 2007), which contains 22 attitudinal items along four subscales, “(Dietary) Restraint”, “Eating Concern”, “Shape Concern”, and “Weight Concern”, that can also be combined into a global score of cognitive and behavioral eating disorders (ED) symptoms over the last 28 days, rated on a 7‐point scale (from 0, never, to 6, every day). Patients further completed the Beck Depression Inventory (BDI), a widely used self‐report inventory, to assess the severity of depressive symptoms at admission and discharge (Hautzinger et al. 1994). The BDI contains 21 items, scored on a 4‐point scale, with sum scores ranging between 0 and 63. Additional questionnaires, not included in the present analysis, are described elsewhere (Halbeisen, Braks, et al. 2022).

The nursing team measured patients' body height (in m) at admission. Body weight (in kg), used for calculating the BMI, was assessed using a calibrated scale (KERN & SOHN GmbH, Balingen‐Frommern, Germany) at admission, daily during the first week after admission, and at least once per week as of week two of treatment until being discharged.

### 2.4 Statistical Analysis

The questionnaires were aggregated according to their convention (for means and SDs, see Table 1). Within‐person BMI gain (∆ BMI) served as our primary dependent variable, which we calculated from the collected weights throughout treatment to reflect changes in BMI from admission. To account for the fact that the measurements occurred with different frequencies during treatment (e.g., weights were measured more frequently at the start of treatment), ∆ BMI values were averaged into a weekly time series. We further standardized ∆ BMI by z‐transforming each value within each patient, which has been shown to improve the estimation of within‐person time‐varying effects (Wang et al. 2019).

**TABLE 1:** Patient sociodemographic and clinical characteristics (means and SDs) at admission.

Variable	Total	Restricting AN	Binge/purge AN	*p*
*n*	518	308	210	
Age (years)	21.8 (8.32)	21.0 (7.02)	23.0 (9.84)	0.03
Age at AN onset	17.0 (5.17)	16.7 (4.49)	17.5 (6.04)	0.20
BMI (kg/m^2^)	15.9 (1.63)	15.7 (1.64)	16.2 (1.57)	< 0.001
Gender: female	504	303	201	0.07
Secondary education				
Highest	140	91	49	0.05
Advanced	23	9	14	
Intermediate	36	15	21	
Lowest	7	5	2	
No degree/pupil	145	90	55	
Missing	167	98	69	
Marital status				
Married	37	15	22	0.18
Single	293	181	112	
Divorced/separated	8	5	3	
No response	11	7	4	
Missing	169	100	69	
Total days hospitalized	63.33 (16.5)	64.56 (17.3)	61.5 (15.1)	0.04
EDE‐Q global score	4.87 (1.22)	4.73 (1.25)	5.09 (1.13)	< 0.001
Restraint	4.91 (1.56)	4.79 (1.63)	5.08 (1.44)	0.04
Eating concern	4.41 (1.27)	4.25 (1.28)	4.64 (1.22)	< 0.001
Weights concern	4.81 (1.45)	4.64 (1.43)	5.07 (1.44)	< 0.001
Shape concern	5.37 (1.37)	5.23 (1.39)	5.57 (1.31)	< 0.006
BDI sum score	27.2 (10.3)	26.3 (10.39)	28.6 (9.96)	0.01

*Note: p*‐values for continuous variables are based on independent *t*‐test, and for categorical variables are based on *χ* ^2^‐test.

Abbreviations: AN = anorexia nervosa; BDI = Beck depression inventory; BMI = body‐mass‐index; EDE‐Q = eating disorder examination‐questionnaire.

With these variables, we conducted two sets of analyses. First, we clustered the standardized ∆ BMI time series using a formulation of DTW with a differentiable loss function, called soft‐DTW (Cuturi and Blondel 2017). The number of clusters (k) was reached by consensus between different cluster validity indices (sum of squared error [SSE] scree (“elbow”) plot, Context‐independent Optimality and Partiality index [COP], Davies–Bouldin index [DB], modified Davies–Bouldin index [DB*]). Other distance functions and centroids were also considered but dismissed due to worse performance across the cluster validity indices (see Supporting Information). Second, we characterized clusters based on the results of univariate comparisons of admission scores (using one‐way ANOVA), and we analyzed associations of cluster identity with changes in clinical outcomes between admission and discharge using linear and logistic models (one‐way ANCOVA, logistic regression), adjusted for the respective admission value and total length of treatment.

The significance level for all analyses was set at *p* ≤ 0.05. Bonferroni adjustment was used for post hoc pairwise comparisons. Variable values are reported as means (M) and standard deviations (SDs). The data were aggregated and analyzed with R 4.1.2 (R Core Team 2022), with package dwclust used for clustering (Sarda‐Espinosa 2024), and TSstudio (Krispin 2020) and tsibble (Wang et al. 2020) for time series preparation.

## 3 Results

### 3.1 Sample Characteristics

The included sample's admission characteristics are summarized in Table 1. Patients with restricting AN, compared to patients with binge‐eating/purging AN, were younger, had a lower BMI, spent more days in treatment, and had lower admission psychopathology scores. Other sociodemographic features were comparable between the AN subtypes.

### 3.2 DTW Clustering and Cluster Comparisons

**FIGURE 2:**
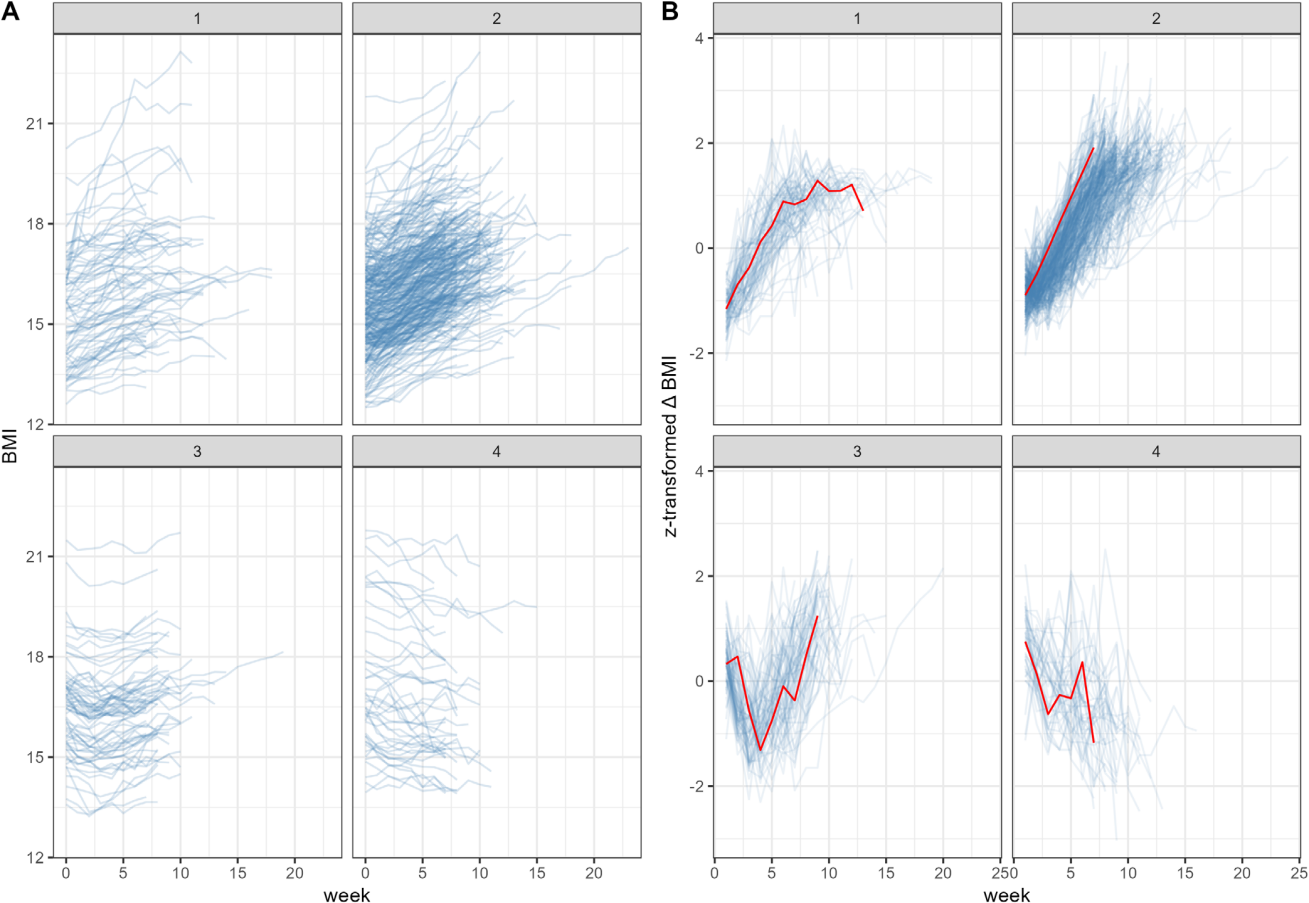
Raw BMI trajectories (A) and standardized ∆ BMI trajectories (B) by SDTW cluster. The red line in (B) marks the most central (and thus most representative) trajectory according to Soft‐DTW centroid function.

Comparisons between the different cluster validity indices (elbow plot, DB, and DB*) suggested k = 4 clusters of standardized ∆ BMI as the optimal solution, see Figure 2, with *n* = 76 patients assigned to Cluster 1 (C1), *n* = 329 to Cluster 2 (C2), *n* = 70 to Cluster 3 (C3), and *n* = 43 to Cluster 4 (C4). The four clusters differed in terms of admission BMI, EDE‐Q scores, and days spent in treatment (see Table 2). C1 and C2 had lower admission BMIs compared to the other clusters; C2 had the lowest EDE‐Q scores, and C4 spent the fewest days in inpatient treatment. All clusters were comparable in age and other sociodemographic features and had similar distributions of AN subtypes; the uncorrelated features were thus not included in further analyses.

**TABLE 2:** Patient Sociodemographic and clinical characteristics (means and SDs) at admission, by clusters.

Variable	C1	C2	C3	C4	*p*
*N*	76	329	70	43	
Age	20.6 (7.63)	21.9 (8.70)	23.6 (8.28)	20.6 (5.71)	0.28
Age at AN onset	16.4 (4.63)	17.0 (5.20)	18.4 (6.27)	16.0 (3.62)	0.20
BMI	15.4 (1.56)	15.7 (1.41)	16.8 (1.57)	17.4 (2.12)	< 0.001
Gender: female	74	319	69	42	0.90
Secondary education					0.25
Highest	19	92	17	12	
Advanced	2	15	3	3	
Intermediate	7	17	10	2	
Lowest	0	7	0	0	
No degree/pupil	24	98	13	10	
Missing	24	100	27	16	
Marital status					0.39
Married	2	26	8	1	
Single	49	187	33	24	
Divorced/separated	0	6	1	1	
No response	1	9	1	0	
Missing	24	101	27	17	
Total days of hospitalization	66.5 (19.2)	64.2 (16.5)	59.9 (14.1)	56.8 (12.8)	0.004
EDEQ global score	5.00 (1.19)	4.72 (1.24)	5.24 (1.10)	5.21 (1.02)	< 0.001
Restraint	5.04 (1.53)	4.74 (1.59)	5.28 (1.44)	5.34 (1.43)	0.009
Eating concern	4.49 (1.23)	4.31 (1.29)	4.67 (1.17)	4.61 (1.23)	0.10
Weights concern	5.00 (1.52)	4.63 (1.45)	5.21 (1.32)	5.23 (1.25)	< 0.001
Shape concern	5.48 (1.30)	5.21 (1.44)	5.81 (1.17)	5.67 (0.99)	0.002
BDI sum score	27.6 (11.1)	26.9 (10.1)	27.7 (9.4)	27.9 (11.8)	0.86
AN subtype					0.35
Restricting (F50.00)	42	205	39	22	
Binge‐eating/purging (F50.01)	34	124	31	21	

*Note: p*‐values for continuous variables are based on ANOVA, and for categorical variables are based on the *χ* ^2^‐test.

Abbreviations: AN = anorexia nervosa; BDI = Beck depression inventory; BMI = body‐mass‐index; EDE‐Q = eating disorder examination‐questionnaire.

Cluster assignment predicted changes in BMI and ED‐specific psychopathology from admission to discharge, controlled for the admission scores and the total length of treatment (see Table 3). C2 showed the largest BMI gains, followed by C1, C3, and C4. Descriptively, C4 lost weight compared to admission. Using C4 as reference, the odds of achieving a healthy BMI ≥ 18.5 kg/m^2^ were also significantly improved in C1 and C2 (overall classification accuracy: 94.6%). Improvements in ED psychopathology, according to EDE‐Q global scores, differed overall, although adjusted pairwise comparisons showed no significant differences between the clusters. However, based on subscale analysis, C2 showed the largest reductions in Eating Concerns compared to C3. General psychopathology, that is, BDI scores, also significantly improved in C2 compared to C1.

**TABLE 3:** Predicted changes in weight‐related outcomes and psychopathology from admission to discharge (means and SEs).

Variable	C1	C2	C3	C4	*p*
*N*	76	329	70	43	
∆ BMI	0.86 (0.09)	1.34 (0.05)	0.14 (0.10)	−0.56 (0.13)	< 0.001
OR BMI ≥ 18.5 kg/m^2^ (%_raw_; %_adj_)	32.9 (9.2; 15.3)	44.8 (12.2; 15.4)	3.3 (11.4; 1.0)	Ref. (21.0; 3.0)	< 0.001
∆ EDE‐Q global score	−1.53 (0.11)	−1.80 (0.05)	−1.52 (0.12)	−1.89 (0.15)	0.03
∆ Restraint	−2.00 (0.16)	−2.28 (0.08)	−1.96 (0.17)	−1.93 (0.21)	0.12
∆ Eating concern	−1.44 (0.12)	−1.71 (0.06)	−1.24 (0.13)	−1.70 (0.16)	0.004
∆ Weights concern	−1.22 (0.14)	−1.51 (0.07)	−1.32 (0.14)	−1.75 (0.18)	0.07
∆ Shape concern	−0.95 (0.14)	−1.21 (0.07)	−1.07 (0.15)	−1.57 (0.19)	0.06
∆ BDI sum score	−8.71 (0.96)	−11.54 (0.46)	−10.70 (1.00)	−9.14 (1.30)	0.03

*Note*: All estimates adjusted for admission value and length of treatment based on ANCOVA or logistic regression (for OR), respectively.

Abbreviations: %_adj_ = predicted % of weight restored patients adjusted for admission differences; %_raw_ = unadjusted % of weight‐restored patients in cluster; BDI = Beck depression inventory; BMI = body‐mass‐index; EDE‐Q = eating disorder examination‐questionnaire; OR = odds ratio; ref. = reference group in logistic regression.

## 4 Discussion

The aim of this study was to innovate on identifying and characterizing individual‐level weight change trajectories among patients with AN during inpatient treatment through DTW‐based time series clustering. Using data from more than 500 patients with AN, we identified four clusters, which fell within the expected range of the previously observed three to five weight gain profiles. Similar to most previous studies, the largest group (C2) included patients with near‐linear weight gain (Di Lodovico et al. 2024; Jennings et al. 2018; Lebow et al. 2019; Makhzoumi et al. 2017). These patients showed continuous improvements in BMI throughout the entirety of their inpatient treatment and had the highest odds of achieving normal weight at discharge. They also showed some of the largest decreases in ED and general psychopathology, suggesting continuous gain as the cluster with the largest overall improvement.

A considerably smaller group (Cluster 1) characterized patients with initial weight gain at treatment onset that reached a plateau toward the end (around 6 weeks, corresponding to the clinic's average length of treatment), again similar to previous observations (Berona et al. 2018; Di Lodovico et al. 2024; Lebow et al. 2019; Makhzoumi et al. 2017; Wade et al. 2021). Some suggest this pattern of a marked initial gain that levels off at a target point as the “textbook treatment response” (Lebow et al. 2019). However, although these patients improved in psychopathology and had high odds of achieving normal discharge weight compared to other groups, we found their improvements to remain lower compared to C2 patients. Thus, one might speculate that these patients needed adjustment in their treatment regimen after showing initial progress and that further improvements might have been achievable.

Contrasting the continuous and initial weight gain clusters, the two remaining clusters were characterized by initial weight loss. C3 included patients with initial weight loss and subsequent recovery that resulted descriptively in net weight gain, whereas C4 patients' BMI declined throughout treatment, resulting in net weight loss. Thus far, similar patterns have only been observed by Jennings et al. (2018) who suggested that weight loss may occur in patients who increased their energy expenditure during treatment and then left prematurely due to experiencing treatment difficulties. However, we must note that C4 patients still showed decreases in ED psychopathology comparable to C2 (continuous gain) while spending the fewest days in treatment, which could suggest that these patients may have concluded treatment prematurely due to additional reasons. It is also plausible that patients in C4 were less motivated to gain weight, given their higher admission BMI, or that in this group (again, due to higher admission BMI), nutritional protocols were less strictly adhered to. The observed improvement in ED psychopathology in the absence of weight gain requires further investigation.

Knowing which patient “phenotype” might show low weight gain or even lose weight at the start of treatment could help inform treatment strategies and allocate therapeutic resources to individual patients more efficiently. After all, AN is expensive to treat (Krauth et al. 2002), and many patients fail to reach weight restoration targets (Zeeck et al. 2018). Similar to previous studies, comparisons based on patient admission characteristics showed that admission BMI and severity of ED psychopathology predicted cluster assignment. Continuous and initial‐only weight gain were associated with a low admission BMI, and low‐ vs. medium‐level severity in ED psychopathology scores distinguished between the groups (Berona et al. 2018; Jennings et al. 2018; Lebow et al. 2019). Higher ED psychopathology scores at admission were associated with initial weight loss (Lebow et al. 2019), with medium vs. high admission body weight distinguishing between groups with and without subsequent recovery (Di Lodovico et al. 2024). In other words, more advantageous weight gain patterns were associated with low BMI and less severe admission psychopathology, whereas the reverse combination of higher BMI with more severe psychopathology predicted low to no weight improvements. Notably, AN subtype did not predict group assignment (Di Lodovico et al. 2024; Makhzoumi et al. 2017), suggesting a more prominent role of overarching diagnostic characteristics in explaining treatment outcomes. Thus, combining features regularly assessed as part of AN treatment, such as admission BMI and global ED psychopathology, might serve as a valuable guide for planning and monitoring a patient's treatment.

Further research is needed to replicate and validate the tentative conclusion regarding the prognostic ability of different weight gain profiles. Still, we can note that the current investigation combines several key strengths. First, we used a novel approach for characterizing weight trajectories that is able to account for similar weight gain profiles stretching over different time periods. This approach replicated some of the key findings of previous (larger) studies while providing an overall more distinct set of meaningful profiles. Second, we analyzed weight trajectories using one of the largest hitherto examined samples, matched only by Jennings et al. with 500 patients (Jennings et al. 2018). The fact that the number and specific profiles found in our study were similar to Jennings et al. further strengthens our approach and its conclusions.

Of course, there are also some limitations. Despite including a large sample of patients, we analyzed their data retrospectively and only included patients who had completed a significant amount of treatment. These patients may have been more compliant or possessed sufficient resources to stay in treatment, which may have skewed part of the results. Further prospective research is thus required. Relatedly, and although representative of many treatment settings, our sample included almost exclusively women. Given that weight gain profiles might differ based on patient gender (Brandt et al. 2023; Halbeisen, Braks, et al. 2024), future prospective studies should aim for the inclusion of a more diverse ED population (Halbeisen, Brandt, et al. 2022). We must also note that the specific treatment setting and therapeutic approaches may limit the generalizability of our findings, and thus also require further investigation. For example, weight trajectories may depend on treatment intensity, discharge criteria, or therapeutic modality, though we were unable to explore such associations using our current data. Finally, by accounting for warped timing dimensions, DTW‐based clustering is inherently oblivious to the absolute speed of weight gain, which has been associated with improved clinical outcomes (Halbeisen, Amin, et al. 2024; Kolar et al. 2022). Thus, linear and non‐linear analysis of weight trajectories should be viewed as complementary.

## 5 Conclusion

Identifying and understanding the heterogeneity in weight restoration has important implications for the treatment of AN. The novel DTW‐based approach used here addressed the discrepancies described in the literature regarding the number and shape of relevant weight gain profiles. This method replicated some of the key findings of previous (larger) studies while providing an overall more distinct set of meaningful weight gain profiles. These results could help inform treatment strategies and allocate therapeutic resources efficiently.

## Author Contributions

**Marianne Tokic:** conceptualization, formal analysis, writing – original draft, writing – review and editing, methodology, visualization. **Georg Halbeisen:** conceptualization, methodology, formal analysis, visualization, writing – original draft, writing – review and editing. **Karsten Braks:** data curation, writing – review and editing. **Thomas J. Huber:** data curation, writing – review and editing. **Nina Timmesfeld:** methodology, supervision, writing – review and editing. **Georgios Paslakis:** conceptualization, supervision, resources, project administration, writing – review and editing.

## Ethics Statement

The study was reviewed and approved by the Ethics Committee of the Ruhr‐University Bochum's Medical Faculty at Campus East‐Westphalia, approval number AZ 2021‐849.

## Consent

Consent for data collection was obtained from all patients within the context of their treatment.

## Conflicts of Interest

The authors declare no conflicts of interest.

## Supporting information


**Data S1:** Supporting Information.

## Data Availability

The data that support the findings of this study are available from the corresponding author upon reasonable request.
