# Supplementary material for: Weight Trajectories During Inpatient Treatment for Anorexia Nervosa: A Dynamic Time Warp Analysis
Source: Int J Eat Disord. 2025 Oct 15;59(1):169–78. doi: 10.1002/eat.24573 (PMC12773659; doi:10.1002/eat.24573)
Supplement: Supplementary file 1 — Data S1: Supporting Information. [file EAT-59-169-s001.docx]

# Online Supplementary Material

## Cluster validity

### 1. Centroid: PAM (Partitioning Around Means), Distance: DTW


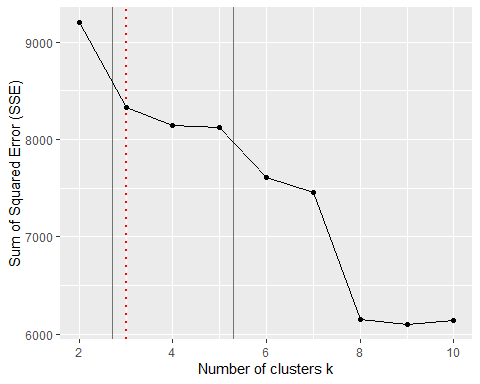


Plot of Sum of Squared Error against potential number of clusters, k (Ellbow-plot). The area within the grey vertical lines is supported by literature (see Introduction). The dashed red line marks the optimal k within this area (“Ellbow”, 3).

| k | Sil | COP | DB | DBstar |
| --- | --- | --- | --- | --- |
| 2 | 0.553284371 | 0.3265385 | 1.319484 | 1.319484 |
| 3 | 0.134117707 | 0.2671595 | 2.251912 | 3.060131 |
| 4 | 0.160091783 | 0.2761634 | 2.291345 | 3.105349 |
| 5 | 0.059704217 | 0.2600423 | 2.720790 | 3.608578 |
| 6 | 0.031875661 | 0.2308689 | 2.699136 | 3.902826 |
| 7 | 0.003462104 | 0.2360761 | 2.016372 | 3.012045 |
| 8 | 0.062651522 | 0.2238009 | 2.007258 | 2.482301 |
| 9 | 0.007213832 | 0.2107356 | 2.153140 | 2.835686 |
| 10 | 0.039214197 | 0.2019466 | 2.442809 | 3.452282 |

Sil = Silhouette index (Rousseeuw (1987)), to be maximized;

COP = COP index (Arbelaitz et al. (2013)), to be minimized);

DB = Davies-Bouldin index (Arbelaitz et al. (2013)), to be minimized;

DBstar = Modified Davies-Bouldin index (DB*) (Kim and Ramakrishna (2005)), to be minimized;

Grey area of table is supported by literature. Red numbers mark optimal values of the indices within this area.

The agreement method suggests **k = 3.**

### 2. Centroid: DTW Barycenter Averaging (DBA), Distance: DTW

As the combination of k and window-size matters in DBA-centering, both have been searched.

## [[1]]
## partitional clustering with 2 clusters
## Using dtw_basic distance
## Using dba centroids
##
## Time required for analysis:
## User System verstrichen
## 30.89 4.02 4.75
##
## Cluster sizes with average intra-cluster distance:
##
## size av_dist
## 1 67 2.072333
## 2 451 1.145970
##
## [[2]]
## partitional clustering with 3 clusters
## Using dtw_basic distance
## Using dba centroids
##
## Time required for analysis:
## User System verstrichen
## 30.89 4.02 4.75
##
## Cluster sizes with average intra-cluster distance:
##
## size av_dist
## 1 155 1.270781
## 2 293 1.011127
## 3 70 1.959637
##
## [[3]]
## partitional clustering with 4 clusters
## Using dtw_basic distance
## Using dba centroids
##
## Time required for analysis:
## User System verstrichen
## 30.89 4.02 4.75
##
## Cluster sizes with average intra-cluster distance:
##
## size av_dist
## 1 180 1.0623343
## 2 230 0.8448804
## 3 38 1.6798272
## 4 70 1.6933160
##
## [[4]]
## partitional clustering with 5 clusters
## Using dtw_basic distance
## Using dba centroids
##
## Time required for analysis:
## User System verstrichen
## 30.89 4.02 4.75
##
## Cluster sizes with average intra-cluster distance:
##
## size av_dist
## 1 41 1.6678358
## 2 50 1.5964794
## 3 175 0.7935934
## 4 139 0.9179590
## 5 113 1.1730189
##
## [[5]]
## partitional clustering with 6 clusters
## Using dtw_basic distance
## Using dba centroids
##
## Time required for analysis:
## User System verstrichen
## 30.89 4.02 4.75
##
## Cluster sizes with average intra-cluster distance:
##
## size av_dist
## 1 79 0.9079538
## 2 92 0.9612599
## 3 194 0.8143824
## 4 37 1.5578873
## 5 55 1.6815118
## 6 61 1.2656578
##
## [[6]]
## partitional clustering with 7 clusters
## Using dtw_basic distance
## Using dba centroids
##
## Time required for analysis:
## User System verstrichen
## 30.89 4.02 4.75
##
## Cluster sizes with average intra-cluster distance:
##
## size av_dist
## 1 52 1.0871946
## 2 35 1.5568101
## 3 158 0.8396463
## 4 40 1.6317576
## 5 125 0.7241300
## 6 51 1.1821758
## 7 57 1.3133352
##
## [[7]]
## partitional clustering with 8 clusters
## Using dtw_basic distance
## Using dba centroids
##
## Time required for analysis:
## User System verstrichen
## 30.89 4.02 4.75
##
## Cluster sizes with average intra-cluster distance:
##
## size av_dist
## 1 29 1.4690120
## 2 20 1.3633944
## 3 21 1.3423240
## 4 161 0.7413319
## 5 29 1.3734619
## 6 83 1.1934280
## 7 143 0.8240724
## 8 32 1.5033653
##
## [[8]]
## partitional clustering with 9 clusters
## Using dtw_basic distance
## Using dba centroids
##
## Time required for analysis:
## User System verstrichen
## 30.89 4.02 4.75
##
## Cluster sizes with average intra-cluster distance:
##
## size av_dist
## 1 37 1.3219570
## 2 115 0.8724547
## 3 32 1.5535101
## 4 67 1.1339969
## 5 17 1.7225108
## 6 114 0.7782703
## 7 83 0.6772343
## 8 26 1.1071520
## 9 27 1.3243616
##
## [[9]]
## partitional clustering with 10 clusters
## Using dtw_basic distance
## Using dba centroids
##
## Time required for analysis:
## User System verstrichen
## 30.89 4.02 4.75
##
## Cluster sizes with average intra-cluster distance:
##
## size av_dist
## 1 28 1.2353277
## 2 34 1.5629934
## 3 27 1.2279362
## 4 38 1.1447373
## 5 35 1.7134394
## 6 113 0.6368280
## 7 42 1.1254725
## 8 91 0.7252893
## 9 45 0.8908468
## 10 65 0.8237126
##
## [[10]]
## partitional clustering with 11 clusters
## Using dtw_basic distance
## Using dba centroids
##
## Time required for analysis:
## User System verstrichen
## 30.89 4.02 4.75
##
## Cluster sizes with average intra-cluster distance:
##
## size av_dist
## 1 53 1.3424337
## 2 27 1.3152043
## 3 61 0.9003729
## 4 29 1.2457889
## 5 11 1.6587798
## 6 23 1.4127142
## 7 80 0.6428106
## 8 48 1.0857483
## 9 59 0.8342985
## 10 116 0.6796245
## 11 11 1.6114528

For each k, the optimal window size was chosen to proceed. All following results built upon that k-window size combination.


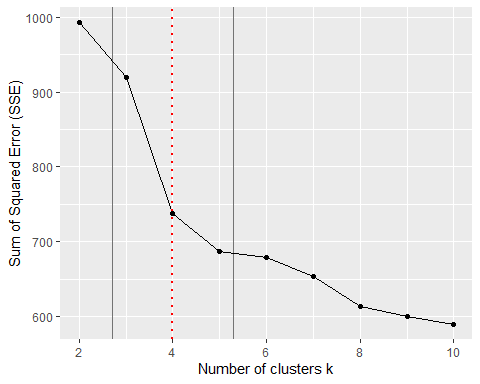


Plot of Sum of Squared Error against potential number of clusters, k (Ellbow-plot). The area within the grey vertical lines is supported by literature (see Introduction). The dashed red line marks the optimal k within this area (“Ellbow”, 4).

| k | Sil | COP | DB | DBstar |
| --- | --- | --- | --- | --- |
| 2 | 0.58962453 | 0.2840590 | 0.9592734 | 0.9592734 |
| 3 | 0.21925301 | 0.2606835 | 1.4966595 | 1.9480407 |
| 4 | 0.20789613 | 0.2104093 | 1.4416526 | 1.8827550 |
| 5 | 0.13577120 | 0.1933835 | 1.7386225 | 2.2594633 |
| 6 | 0.14491954 | 0.2009672 | 1.6233214 | 2.1291089 |
| 7 | 0.10031383 | 0.1840318 | 1.6951360 | 2.1832330 |
| 8 | 0.12311672 | 0.1875405 | 1.8592461 | 2.1797367 |
| 9 | 0.08671588 | 0.1800705 | 1.6550129 | 2.2270461 |
| 10 | 0.09110588 | 0.1721142 | 1.7529775 | 2.5439777 |

Sil = Silhouette index (Rousseeuw (1987)), to be maximized;

COP = COP index (Arbelaitz et al. (2013)), to be minimized);

DB = Davies-Bouldin index (Arbelaitz et al. (2013)), to be minimized;

DBstar = Modified Davies-Bouldin index (DB*) (Kim and Ramakrishna (2005)), to be minimized;

Grey area of table is supported by literature. Red numbers mark optimal values of the indices within this area.

The agreement method suggests **k = 4.**

### 3. Centroid: soft DTW, Distance: soft DTW


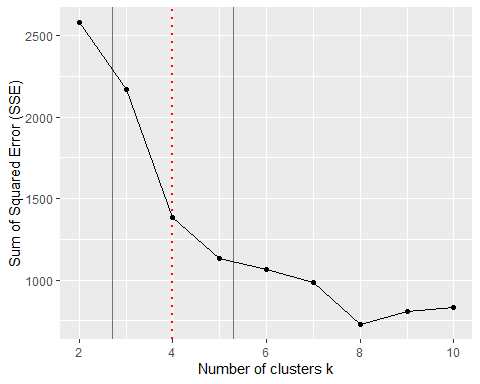


Plot of Sum of Squared Error against potential number of clusters, k (Ellbow-plot). The area within the grey vertical lines is supported by literature (see Introduction). The dashed red line marks the optimal k within this area (“Ellbow”, 4).

| k | Sil | COP | DB | DBstar |
| --- | --- | --- | --- | --- |
| 2 | 0.7953422 | 0.09874874 | 0.5083151 | 0.5083151 |
| 3 | 0.3644363 | 0.08623152 | 1.2122496 | 2.4432466 |
| 4 | 0.3553969 | 0.05532443 | 1.2026790 | 2.5375644 |
| 5 | 0.3275092 | 0.04664166 | 1.5310280 | 2.9228395 |
| 6 | 0.1928446 | 0.04547609 | 1.5216029 | 2.9726918 |
| 7 | 0.2224049 | 0.04144762 | 1.3211574 | 3.0688246 |
| 8 | 0.2414338 | 0.03759106 | 1.7222341 | 2.8147847 |
| 9 | 0.1080694 | 0.03897150 | 1.5869929 | 3.6741820 |
| 10 | 0.1522872 | 0.03540604 | 1.4675363 | 3.7373598 |

Sil = Silhouette index (Rousseeuw (1987)), to be maximized;

COP = COP index (Arbelaitz et al. (2013)), to be minimized);

DB = Davies-Bouldin index (Arbelaitz et al. (2013)), to be minimized;

DBstar = Modified Davies-Bouldin index (DB*) (Kim and Ramakrishna (2005)), to be minimized;

Grey area of table is supported by literature. Red numbers mark optimal values of the indices within this area.

The agreement method suggests both **k = 3 and k= 4** to be good candidates. As the one diverging metric (COP) suggests a higher number and Sil and DB* show neglible differences between k = 3 and k = 4, **the higher cluster number (k = 4) is chosen**.

## Comparison of cluster methods


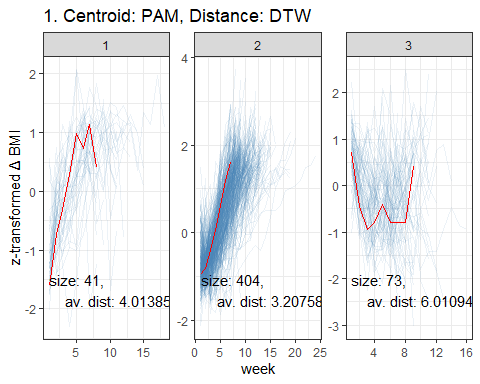


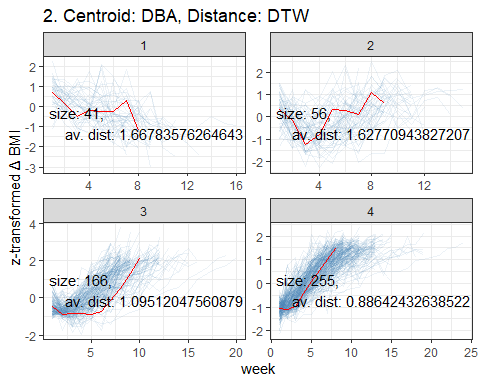


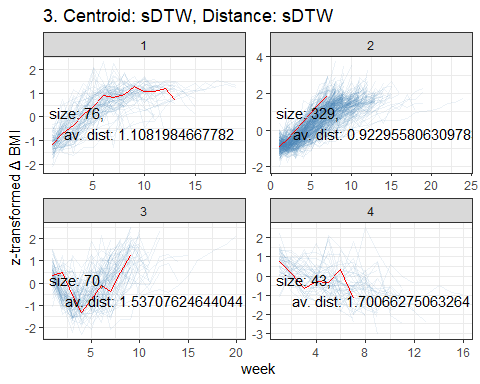


| k | Sil | COP | DB | DBstar | method |
| --- | --- | --- | --- | --- | --- |
| 3 | 0.1341177 | 0.26715948 | 2.251912 | 3.060131 | PAM DTW |
| 4 | 0.2078961 | 0.21040932 | 1.441653 | 1.882755 | DBA DTW |
| 4 | 0.3553969 | 0.05532443 | 1.202679 | 2.537564 | sDTW |

Sil = Silhouette index (Rousseeuw (1987)), to be maximized;

COP = COP index (Arbelaitz et al. (2013)), to be minimized);

DB = Davies-Bouldin index (Arbelaitz et al. (2013)), to be minimized;

DBstar = Modified Davies-Bouldin index (DB*) (Kim and Ramakrishna (2005)), to be minimized.

### Conclusion

The PAM method ranks the lowest of all tried methods across all indices. It delivers extreme cluster sizes and preforms worst across all validity indices used. Both the sDTW and the DBA DTW method deliver clusters of similar quality, and comparison of the centroid plots reveals a high agreement between the methods in terms of content. The sDTW clustering has a clear advantage in the SIL, COP, and DB indices, while only preforming worse according to the DB* index. Additionally, the sDTW method relies on one less parameter to optimize, making it the more parsimonious choice.

All in all, **the sDTW clustering method is chosen for the main analysis**.

## Literature mentioned in Online Supplementary Material

1.: Rousseeuw, P. J. (1987). Silhouettes: a graphical aid to the interpretation and validation of cluster analysis. Journal of computational and applied mathematics, 20, 53-65.

2.: Arbelaitz, O., Gurrutxaga, I., Muguerza, J., Perez, J. M., & Perona, I. (2013). An extensive comparative study of cluster validity indices. Pattern Recognition, 46(1), 243-256.

3.: Kim, M., & Ramakrishna, R. S. (2005). New indices for cluster validity assessment. Pattern Recognition Letters, 26(15), 2353-2363.
